# Poly[tetra­aqua­bis­(μ_4_-thio­phene-2,5-dicarboxyl­ato)(μ_2_-thio­phene-2,5-dicarboxyl­ato)dieuropium(III)]

**DOI:** 10.1107/S1600536812029546

**Published:** 2012-07-04

**Authors:** Xi-Gang Du, Jun Zhang, Jia-Jia Li

**Affiliations:** a School of Chemical Engineering and Pharmaceutics, Henan University of Science and Technology, Luoyang 471003, People’s Republic of China; b Department of Chemistry, Zhengzhou University, Zhengzhou 450001, People’s Republic of China

## Abstract

The three-dimensional coordination polymer, [Eu_2_(C_6_H_2_O_4_S)_3_(H_2_O)_4_]_*n*_, has been synthesized under hydro­thermal conditions. The asymmetric unit comprises one Eu^3+^ cation, two aqua ligands and one and a half thiophene-2,5-dicarboxylate anions (the half-anion being completed by a twofold rotation axis). The Eu^3+^ cation is eight-coordinated in a distorted dodeca­hedral geometry. The crystal structure features O—H⋯O hydrogen bonds.

## Related literature

For the structures and potential applications of metal hybrid compounds, see: Bo *et al.* (2008 ▶). For a number of lanthanide coordination polymers based on pyridine­dicarb­oxy­lic acid, see: Xu *et al.* (2011 ▶). For metal-organic framework structures formed by 4*f* metals and thiophene-2,5-dicarboxylate anions, see: Huang *et al.* (2009 ▶). 
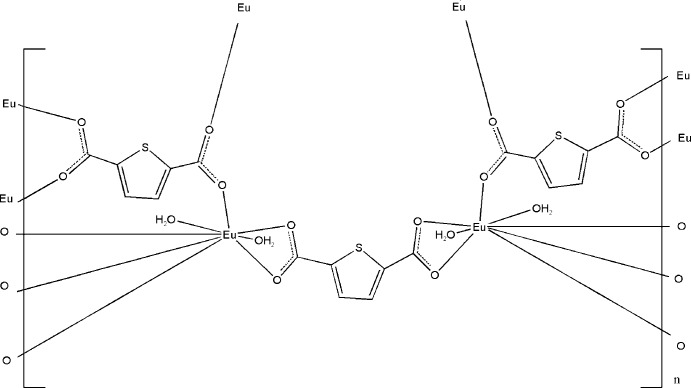


## Experimental

### 

#### Crystal data

[Eu_2_(C_6_H_2_O_4_S)_3_(H_2_O)_4_]
*M* *_r_* = 886.44
Monoclinic, 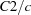

*a* = 25.366 (8) Å
*b* = 5.8326 (14) Å
*c* = 19.008 (6) Å
β = 124.136 (4)°
*V* = 2327.7 (12) Å^3^
*Z* = 4
Mo *K*α radiation
μ = 5.69 mm^−1^
*T* = 295 K
0.22 × 0.12 × 0.11 mm

#### Data collection

Bruker SMART APEXII CCD diffractometer
Absorption correction: multi-scan (*SADABS*; Bruker, 2004 ▶) *T*_min_ = 0.445, *T*_max_ = 0.535
8572 measured reflections
2634 independent reflections
2290 reflections with *I* > 2σ(*I*)
*R*_int_ = 0.061

#### Refinement

*R*[*F* ^2^ > 2σ(*F* ^2^)] = 0.035
*wR*(*F* ^2^) = 0.090
*S* = 1.00
2634 reflections
180 parameters
H atoms treated by a mixture of independent and constrained refinement
Δρ_max_ = 1.97 e Å^−3^
Δρ_min_ = −1.74 e Å^−3^

### 

Data collection: *APEX2* (Bruker, 2004 ▶); cell refinement: *SAINT* (Bruker, 2004 ▶); data reduction: *SAINT*; program(s) used to solve structure: *SHELXS97* (Sheldrick, 2008 ▶); program(s) used to refine structure: *SHELXL97* (Sheldrick, 2008 ▶); molecular graphics: *DIAMOND* (Brandenburg, 1999 ▶); software used to prepare material for publication: *enCIFer* (Allen *et al.*, 2004) ▶ and *PLATON* (Spek, 2009 ▶).

## Supplementary Material

Crystal structure: contains datablock(s) I, global. DOI: 10.1107/S1600536812029546/rk2364sup1.cif

Structure factors: contains datablock(s) I. DOI: 10.1107/S1600536812029546/rk2364Isup2.hkl

Additional supplementary materials:  crystallographic information; 3D view; checkCIF report

## Figures and Tables

**Table 1:** Hydrogen-bond geometry (Å, °)

*D*—H⋯*A*	*D*—H	H⋯*A*	*D*⋯*A*	*D*—H⋯*A*
O7—H7*A*⋯O4^i^	0.85	2.11	2.915 (6)	158
O7—H7*A*⋯O3^ii^	0.85	2.53	3.073 (5)	123
O7—H7*B*⋯O5^ii^	0.85	2.03	2.833 (5)	158
O8—H8*B*⋯O6^iii^	0.85	2.10	2.846 (5)	147
O8—H8*A*⋯O5^iv^	0.85	2.46	2.919 (5)	115

Symmetry codes: (i) 
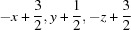
; (ii) 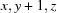; (iii) 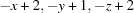; (iv) 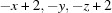.

**Fig. 1.**
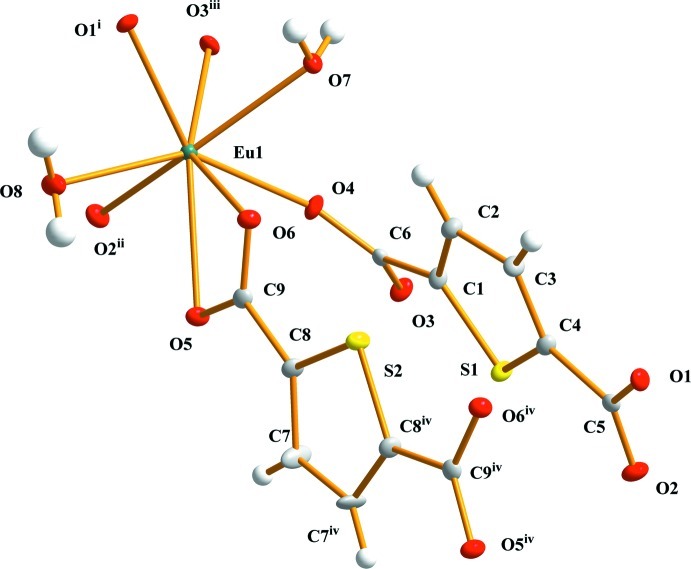
The coordination environment of the Eu3+ with the atom numbering scheme. Displacement ellipsoids are presented at 30% probability level. Symmetry codes: (i) x, 1-y, 1/2+z; (ii) x, -y, 1/2+z; (iii) 3/2-x, 1/2+y, 3/2-z; (iv) 2-x, y, 1/2-z;

**Fig. 2.**
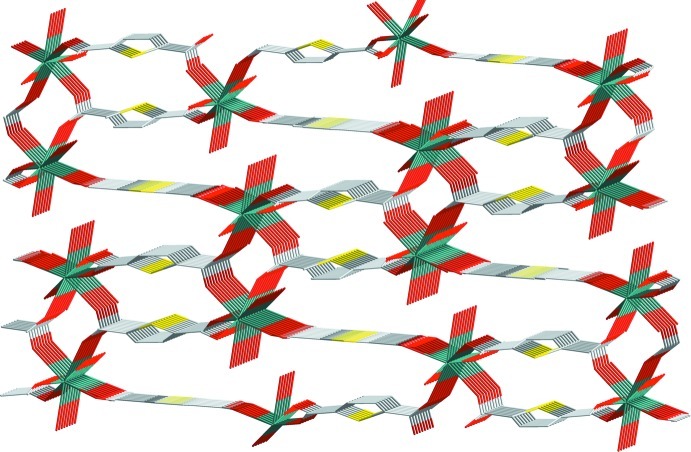
Three-dimensional architecture in the crystal structure of title compound. All the hydrogen atoms are omitted for clarity.

## supplementary crystallographic information

### Comment

The design and synthesis of metal-hybrid compounds have attracted considerable interest due to their intriguing topological structures and potential applications as functional materials in luminescence, magnetism, host-guest chemistry, catalysis and gas adsorption and separation (Bo *et al.*, 2008). In recent years, a number of lanthanide coordination polymers based on pyridinedicarboxylic acid have been synthesized under hydrothermal conditions (Xu *et al.*, 2011). By contrast with these lanthanide complexes containing only rigid pyridinedicarboxylic ligands, the high-dimensional coordination complexes of 4*f* metal-organic frameworks formed by H_2_*tdc* (thiophene-2,5-dicarboxylic acid) are still scarce (Huang *et al.*, 2009). Herein, we report a new structure derived from thiophene-2,5-dicarboxylic acid (Scheme 1), namely [Eu_2_(*tdc*)_3_(H_2_O)_4_]_n_.

A view of the coordination environment of the Eu^3+^ with atom labeling is illustrated in Fig. 1. The Hydrogen-bond are listed in Table 1. The Eu—O bond lengths range from 2.336 (2)Å to 2.493 (1)Å, and the bond angles of O—Eu—O are in the range of 51.87 (11)° to 156.27 (13)°. In structure, *tdc* ligand adopt two different coordination modes, constructing an ordered three-dimensional lanthanide framework (Fig. 2).

### Experimental

All chemicals and solvents except Eu(NO_3_)_3_ were purchased and used as received without further purification. Eu(NO_3_)_3_ was prepared by dissolving Eu_2_O_3_ with concentrated HNO_3_ and then evaporating at 373 K until crystal film formed. A mixture of Eu(NO_3_)_3_ (0.3 mmol), KSCN (0.15 mmol), H_2_*tdc* (0.3 mmol) and deionized water (8.0 ml) in a 23 ml teflon-lined autoclave and kept under autogenous pressure at 443 K for 5 days and then cooling to room temperature at a rate of 5 K h^-1^. Colourless crystals were isolated by filtration.

### Refinement

All hydrogen atoms were positioned geometrically and treated as riding, with C–H = 0.93Å (CH) and *U*_iso_(H) = 1.2*U*_eq_(C), with C–H = 0.97Å (CH_2_) and *U*_iso_(H) = 1.2*U*_eq_(C), and with C–H = 0.96Å (CH_3_) and *U*_iso_(H) = 1.5*U*_eq_(C)

### Crystal data

**Table:**

[Eu_2_(C_6_H_2_O_4_S)_3_(H_2_O)_4_]	*F*(000) = 1696
*M**_r_* = 886.44	*D*_x_ = 2.530 Mg m^−^^3^
Monoclinic, *C*2/*c*	Mo *K*α radiation, λ = 0.71073 Å
Hall symbol: -C 2yc	Cell parameters from 8572 reflections
*a* = 25.366 (8) Å	θ = 2.6–27.5°
*b* = 5.8326 (14) Å	µ = 5.69 mm^−^^1^
*c* = 19.008 (6) Å	*T* = 295 K
β = 124.136 (4)°	Block, colourless
*V* = 2327.7 (12) Å^3^	0.22 × 0.12 × 0.11 mm
*Z* = 4	

### Data collection

**Table:**

Bruker SMART APEXII CCD diffractometer	2634 independent reflections
Radiation source: fine-focus sealed tube	2290 reflections with *I* > 2σ(*I*)
Graphite monochromator	*R*_int_ = 0.061
φ and ω scans	θ_max_ = 27.5°, θ_min_ = 2.6°
Absorption correction: multi-scan (*SADABS*; Bruker, 2004)	*h* = −32→32
*T*_min_ = 0.445, *T*_max_ = 0.535	*k* = −7→7
8572 measured reflections	*l* = −24→24

### Refinement

**Table:**

Refinement on *F*^2^	Primary atom site location: structure-invariant direct methods
Least-squares matrix: full	Secondary atom site location: difference Fourier map
*R*[*F*^2^ > 2σ(*F*^2^)] = 0.035	Hydrogen site location: inferred from neighbouring sites
*wR*(*F*^2^) = 0.090	H atoms treated by a mixture of independent and constrained refinement
*S* = 1.00	*w* = 1/[σ^2^(*F*_o_^2^) + (0.0272*P*)^2^] where *P* = (*F*_o_^2^ + 2*F*_c_^2^)/3
2634 reflections	(Δ/σ)_max_ = 0.026
180 parameters	Δρ_max_ = 1.97 e Å^−^^3^
0 restraints	Δρ_min_ = −1.74 e Å^−^^3^

### Special details

**Table:**

Geometry. All s.u.'s (except the s.u. in the dihedral angle between two l.s. planes) are estimated using the full covariance matrix. The cell s.u.'s are taken into account individually in the estimation of s.u.'s in distances, angles and torsion angles; correlations between s.u.'s in cell parameters are only used when they are defined by crystal symmetry. An approximate (isotropic) treatment of cell s.u.'s is used for estimating s.u.'s involving l.s. planes.
Refinement. Refinement of *F*^2^ against ALL reflections. The weighted *R*-factor *wR* and goodness of fit *S* are based on *F*^2^, conventional *R*-factors *R* are based on *F*, with *F* set to zero for negative *F*^2^. The threshold expression of *F*^2^ > σ(*F*^2^) is used only for calculating *R*-factors(gt) *etc*. and is not relevant to the choice of reflections for refinement. *R*-factors based on *F*^2^ are statistically about twice as large as those based on *F*, and *R*-factors based on ALL data will be even larger.

### Fractional atomic coordinates and isotropic or equivalent isotropic displacement parameters (Å^2^)

**Table:**

	*x*	*y*	*z*	*U*_iso_*/*U*_eq_	
Eu1	0.866707 (10)	0.33845 (4)	0.887181 (14)	0.01268 (11)	
S1	0.80798 (6)	0.0059 (2)	0.56308 (7)	0.0174 (3)	
S2	1.0000	0.3059 (3)	0.7500	0.0186 (4)	
O1	0.89384 (19)	0.3300 (5)	0.4736 (2)	0.0174 (8)	
O2	0.84987 (18)	−0.0175 (6)	0.4448 (2)	0.0222 (8)	
O3	0.73304 (15)	−0.0491 (6)	0.6396 (2)	0.0183 (7)	
O4	0.79280 (18)	0.1905 (6)	0.7483 (2)	0.0197 (8)	
O5	0.92823 (17)	0.0273 (6)	0.8657 (2)	0.0188 (7)	
O6	0.94444 (17)	0.3894 (6)	0.8480 (2)	0.0186 (7)	
O7	0.83645 (19)	0.6714 (5)	0.7928 (2)	0.0175 (8)	
H7A	0.7974	0.7025	0.7705	0.026*	
H7B	0.8592	0.7858	0.8214	0.026*	
O8	0.96903 (18)	0.2934 (7)	1.0254 (2)	0.0254 (9)	
H8B	0.9831	0.4248	1.0475	0.038*	
H8A	0.9956	0.2279	1.0181	0.038*	
C1	0.8071 (2)	0.1795 (8)	0.6351 (3)	0.0144 (10)	
C2	0.8346 (2)	0.3894 (9)	0.6426 (3)	0.0185 (10)	
H2	0.8382	0.5055	0.6786	0.022*	
C3	0.8565 (2)	0.4072 (9)	0.5900 (3)	0.0171 (10)	
H3	0.8758	0.5383	0.5866	0.021*	
C4	0.8467 (2)	0.2123 (9)	0.5438 (3)	0.0154 (10)	
C5	0.8649 (2)	0.1712 (7)	0.4835 (3)	0.0135 (10)	
C6	0.7759 (2)	0.1015 (8)	0.6773 (3)	0.0139 (9)	
C7	0.9866 (3)	−0.1143 (10)	0.7733 (4)	0.0283 (13)	
C8	0.9766 (2)	0.1034 (9)	0.7912 (3)	0.0172 (10)	
C9	0.9486 (2)	0.1754 (8)	0.8384 (3)	0.0153 (11)	
H7	0.976 (3)	−0.227 (10)	0.794 (3)	0.018*	

### Atomic displacement parameters (Å^2^)

**Table:**

	*U*^11^	*U*^22^	*U*^33^	*U*^12^	*U*^13^	*U*^23^
Eu1	0.01266 (17)	0.01346 (19)	0.01289 (17)	0.00158 (7)	0.00776 (14)	0.00075 (7)
S1	0.0208 (6)	0.0165 (6)	0.0195 (6)	−0.0051 (5)	0.0142 (5)	−0.0040 (5)
S2	0.0227 (9)	0.0160 (8)	0.0265 (9)	0.000	0.0195 (8)	0.000
O1	0.020 (2)	0.020 (2)	0.0183 (19)	−0.0001 (13)	0.0144 (18)	0.0045 (13)
O2	0.0246 (19)	0.0203 (19)	0.0277 (18)	−0.0023 (15)	0.0183 (17)	−0.0077 (15)
O3	0.0154 (16)	0.0226 (18)	0.0193 (16)	−0.0090 (14)	0.0112 (15)	−0.0068 (15)
O4	0.023 (2)	0.026 (2)	0.0136 (17)	−0.0048 (14)	0.0122 (17)	−0.0045 (14)
O5	0.0225 (18)	0.0167 (18)	0.0263 (18)	0.0018 (14)	0.0193 (16)	0.0025 (15)
O6	0.0195 (18)	0.0171 (18)	0.0240 (18)	0.0007 (14)	0.0150 (16)	−0.0003 (15)
O7	0.019 (2)	0.016 (2)	0.0163 (18)	0.0006 (12)	0.0093 (17)	0.0015 (12)
O8	0.0196 (19)	0.0235 (18)	0.0209 (19)	0.0019 (15)	0.0039 (17)	−0.0051 (16)
C1	0.012 (2)	0.018 (3)	0.014 (2)	−0.0042 (17)	0.007 (2)	−0.0008 (17)
C2	0.020 (3)	0.019 (2)	0.018 (2)	−0.002 (2)	0.011 (2)	−0.003 (2)
C3	0.022 (3)	0.013 (2)	0.020 (2)	−0.0036 (19)	0.013 (2)	0.000 (2)
C4	0.012 (2)	0.020 (2)	0.016 (2)	−0.0009 (19)	0.009 (2)	−0.001 (2)
C5	0.010 (2)	0.015 (3)	0.014 (2)	0.0018 (16)	0.006 (2)	0.0016 (17)
C6	0.012 (2)	0.016 (2)	0.013 (2)	0.0052 (19)	0.0073 (19)	0.0038 (19)
C7	0.050 (4)	0.016 (2)	0.044 (3)	−0.008 (3)	0.042 (3)	−0.002 (3)
C8	0.018 (2)	0.020 (2)	0.022 (2)	0.001 (2)	0.016 (2)	0.000 (2)
C9	0.010 (2)	0.020 (3)	0.015 (2)	−0.0005 (17)	0.006 (2)	−0.0012 (18)

### Geometric parameters (Å, º)

**Table:**

Eu1—O2^i^	2.326 (3)	O4—C6	1.275 (6)
Eu1—O1^ii^	2.375 (3)	O5—C9	1.259 (6)
Eu1—O3^iii^	2.376 (3)	O6—C9	1.274 (5)
Eu1—O4	2.382 (4)	O7—H7A	0.8500
Eu1—O7	2.456 (3)	O7—H7B	0.8500
Eu1—O8	2.461 (4)	O8—H8B	0.8500
Eu1—O6	2.486 (4)	O8—H8A	0.8501
Eu1—O5	2.572 (3)	C1—C2	1.376 (7)
S1—C1	1.713 (5)	C1—C6	1.480 (7)
S1—C4	1.718 (5)	C2—C3	1.392 (7)
S2—C8^iv^	1.697 (5)	C2—H2	0.9300
S2—C8	1.697 (5)	C3—C4	1.372 (7)
O1—C5	1.260 (6)	C3—H3	0.9300
O1—Eu1^v^	2.375 (3)	C4—C5	1.476 (7)
O2—C5	1.258 (5)	C7—C8	1.373 (8)
O2—Eu1^vi^	2.326 (3)	C7—C7^iv^	1.389 (11)
O3—C6	1.262 (6)	C7—H7	0.87 (6)
O3—Eu1^vii^	2.376 (3)	C8—C9	1.484 (7)
			
O2^i^—Eu1—O1^ii^	112.82 (13)	C9—O6—Eu1	94.0 (3)
O2^i^—Eu1—O3^iii^	82.43 (12)	Eu1—O7—H7A	109.4
O1^ii^—Eu1—O3^iii^	77.54 (13)	Eu1—O7—H7B	109.4
O2^i^—Eu1—O4	89.55 (13)	H7A—O7—H7B	109.5
O1^ii^—Eu1—O4	143.48 (13)	Eu1—O8—H8B	109.3
O3^iii^—Eu1—O4	77.34 (13)	Eu1—O8—H8A	109.3
O2^i^—Eu1—O7	156.27 (13)	H8B—O8—H8A	109.5
O1^ii^—Eu1—O7	73.27 (14)	C2—C1—C6	127.7 (5)
O3^iii^—Eu1—O7	76.49 (12)	C2—C1—S1	112.1 (4)
O4—Eu1—O7	75.39 (12)	C6—C1—S1	120.2 (3)
O2^i^—Eu1—O8	76.92 (13)	C1—C2—C3	112.0 (5)
O1^ii^—Eu1—O8	68.02 (13)	C1—C2—H2	124.0
O3^iii^—Eu1—O8	128.08 (13)	C3—C2—H2	124.0
O4—Eu1—O8	147.93 (13)	C4—C3—C2	113.4 (5)
O7—Eu1—O8	125.05 (13)	C4—C3—H3	123.3
O2^i^—Eu1—O6	128.58 (12)	C2—C3—H3	123.3
O1^ii^—Eu1—O6	98.00 (13)	C3—C4—C5	127.6 (5)
O3^iii^—Eu1—O6	146.20 (12)	C3—C4—S1	111.3 (4)
O4—Eu1—O6	88.55 (12)	C5—C4—S1	121.1 (4)
O7—Eu1—O6	70.23 (13)	O2—C5—O1	124.5 (5)
O8—Eu1—O6	78.11 (13)	O2—C5—C4	118.1 (4)
O2^i^—Eu1—O5	78.18 (12)	O1—C5—C4	117.3 (4)
O1^ii^—Eu1—O5	135.91 (12)	O3—C6—O4	123.7 (5)
O3^iii^—Eu1—O5	145.99 (12)	O3—C6—C1	117.3 (4)
O4—Eu1—O5	74.85 (12)	O4—C6—C1	119.0 (4)
O7—Eu1—O5	114.24 (12)	C8—C7—C7^iv^	112.4 (3)
O8—Eu1—O5	73.97 (12)	C8—C7—H7	116 (4)
O6—Eu1—O5	51.87 (11)	C7^iv^—C7—H7	131 (4)
C1—S1—C4	91.2 (2)	C7—C8—C9	128.9 (5)
C8^iv^—S2—C8	91.8 (4)	C7—C8—S2	111.7 (4)
C5—O1—Eu1^v^	137.3 (3)	C9—C8—S2	119.4 (4)
C5—O2—Eu1^vi^	154.6 (3)	O5—C9—O6	121.7 (5)
C6—O3—Eu1^vii^	142.1 (3)	O5—C9—C8	120.1 (4)
C6—O4—Eu1	155.3 (3)	O6—C9—C8	118.1 (4)
C9—O5—Eu1	90.4 (3)		
			
O2^i^—Eu1—O4—C6	−109.1 (8)	C1—S1—C4—C5	179.0 (4)
O1^ii^—Eu1—O4—C6	121.1 (8)	Eu1^vi^—O2—C5—O1	40.7 (11)
O3^iii^—Eu1—O4—C6	168.6 (8)	Eu1^vi^—O2—C5—C4	−140.3 (6)
O7—Eu1—O4—C6	89.5 (8)	Eu1^v^—O1—C5—O2	91.7 (6)
O8—Eu1—O4—C6	−45.1 (9)	Eu1^v^—O1—C5—C4	−87.3 (6)
O6—Eu1—O4—C6	19.5 (8)	C3—C4—C5—O2	−177.7 (5)
O5—Eu1—O4—C6	−31.2 (8)	S1—C4—C5—O2	1.7 (7)
O2^i^—Eu1—O5—C9	−174.6 (3)	C3—C4—C5—O1	1.4 (8)
O1^ii^—Eu1—O5—C9	−63.9 (3)	S1—C4—C5—O1	−179.2 (4)
O3^iii^—Eu1—O5—C9	128.8 (3)	Eu1^vii^—O3—C6—O4	36.1 (8)
O4—Eu1—O5—C9	92.6 (3)	Eu1^vii^—O3—C6—C1	−142.9 (4)
O7—Eu1—O5—C9	26.7 (3)	Eu1—O4—C6—O3	140.6 (6)
O8—Eu1—O5—C9	−95.0 (3)	Eu1—O4—C6—C1	−40.4 (10)
O6—Eu1—O5—C9	−7.7 (3)	C2—C1—C6—O3	152.4 (5)
O2^i^—Eu1—O6—C9	24.1 (3)	S1—C1—C6—O3	−24.0 (6)
O1^ii^—Eu1—O6—C9	151.9 (3)	C2—C1—C6—O4	−26.7 (8)
O3^iii^—Eu1—O6—C9	−128.6 (3)	S1—C1—C6—O4	156.9 (4)
O4—Eu1—O6—C9	−64.1 (3)	C7^iv^—C7—C8—C9	179.4 (7)
O7—Eu1—O6—C9	−139.1 (3)	C7^iv^—C7—C8—S2	0.3 (9)
O8—Eu1—O6—C9	86.5 (3)	C8^iv^—S2—C8—C7	−0.1 (3)
O5—Eu1—O6—C9	7.7 (3)	C8^iv^—S2—C8—C9	−179.3 (5)
C4—S1—C1—C2	1.0 (4)	Eu1—O5—C9—O6	14.0 (5)
C4—S1—C1—C6	177.9 (4)	Eu1—O5—C9—C8	−164.0 (4)
C6—C1—C2—C3	−176.9 (5)	Eu1—O6—C9—O5	−14.6 (5)
S1—C1—C2—C3	−0.2 (6)	Eu1—O6—C9—C8	163.5 (4)
C1—C2—C3—C4	−1.0 (7)	C7—C8—C9—O5	−2.5 (8)
C2—C3—C4—C5	−178.9 (5)	S2—C8—C9—O5	176.5 (4)
C2—C3—C4—S1	1.7 (6)	C7—C8—C9—O6	179.4 (6)
C1—S1—C4—C3	−1.5 (4)	S2—C8—C9—O6	−1.6 (6)

Symmetry codes: (i) *x*, −*y*, *z*+1/2; (ii) *x*, −*y*+1, *z*+1/2; (iii) −*x*+3/2, *y*+1/2, −*z*+3/2; (iv) −*x*+2, *y*, −*z*+3/2; (v) *x*, −*y*+1, *z*−1/2; (vi) *x*, −*y*, *z*−1/2; (vii) −*x*+3/2, *y*−1/2, −*z*+3/2.

### Hydrogen-bond geometry (Å, º)

**Table:**

*D*—H···*A*	*D*—H	H···*A*	*D*···*A*	*D*—H···*A*
O7—H7*A*···O4^iii^	0.85	2.11	2.915 (6)	158
O7—H7*A*···O3^viii^	0.85	2.53	3.073 (5)	123
O7—H7*B*···O5^viii^	0.85	2.03	2.833 (5)	158
O8—H8*B*···O6^ix^	0.85	2.10	2.846 (5)	147
O8—H8*A*···O5^x^	0.85	2.46	2.919 (5)	115

Symmetry codes: (iii) −*x*+3/2, *y*+1/2, −*z*+3/2; (viii) *x*, *y*+1, *z*; (ix) −*x*+2, −*y*+1, −*z*+2; (x) −*x*+2, −*y*, −*z*+2.
